# Supplementary material for: Phenotypically complex living materials containing engineered cyanobacteria
Source: Nat Commun. 2023 Aug 7;14:4742. doi: 10.1038/s41467-023-40265-2 (PMC10406891; doi:10.1038/s41467-023-40265-2)
Supplement: Supplementary file 1 — Supplementary Information [file 41467_2023_40265_MOESM1_ESM.pdf]

Supplementary Information

Phenotypically Complex Living Materials Containing Engineered Cyanobacteria

Authors

Debika Datta[1]*, Elliot L. Weiss[2,3]*, Daniel Wangpraseurt[1,2], Erica Hild[1], Shaochen Chen[1], James W. Golden[3], Susan S. Golden[3], Jonathan K. Pokorski[1,4]

* Indicates equal contributions

Author Affiliations

1. *Department of Nanoengineering, University of California San Diego, La Jolla, California, USA*
2. *Scripps Institution of Oceanography, University of California San Diego, La Jolla, California, USA*
3. *Department of Molecular Biology, University of California San Diego, La Jolla, California, USA*
4. *Center for Nano-ImmunoEngineering and Institute for Materials Discovery and Design, University of California San Diego, La Jolla, California, USA*

Files included in this document: Supplementary Figures 1-16.
Separate SI excel sheet given for Supplementary Data 1-4.
Separate SI given for Supplementary Movie 1.

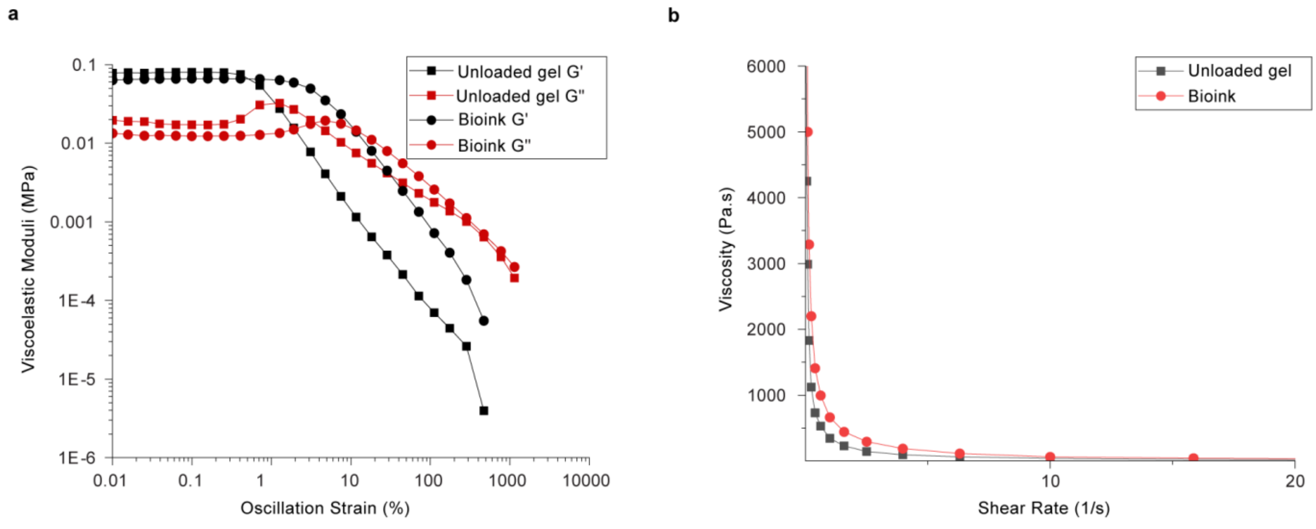

Supplementary Figure 1. Rheological characterization of the unloaded gel and bioink after five days of incubation. a) Viscoelastic moduli vs. oscillatory strain at ω = 10 rad/s and 25 °C. Storage modulus is denoted by G' and loss modulus by G". b) Viscosity vs. shear rate of hydrogels at 25 °C.

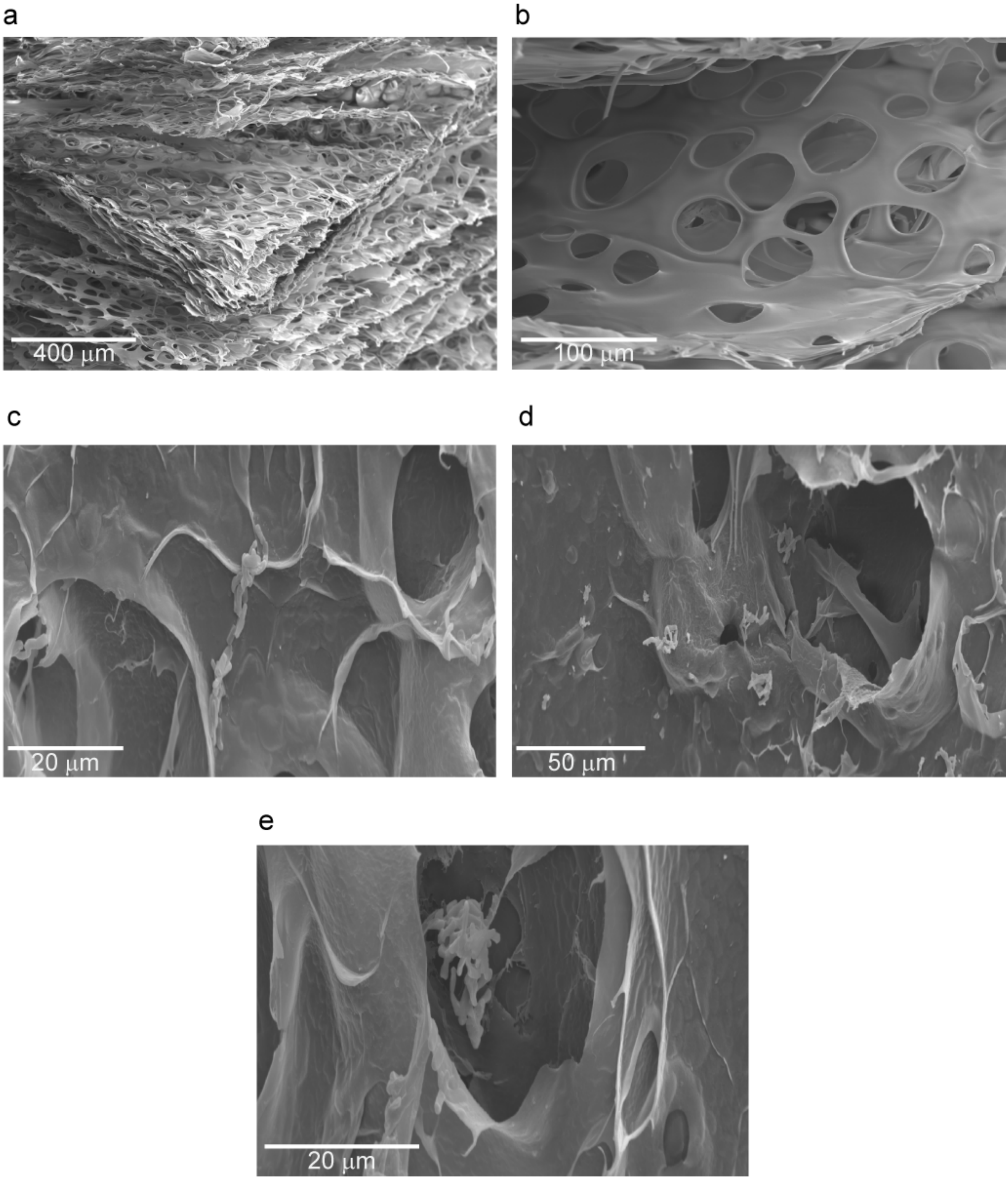

Supplementary Figure 2. Larger sized version of FESEM images presented in Figure 2b of unloaded hydrogels (a & b), and hydrogels containing WT *S. elongatus* cells (c, d and e).

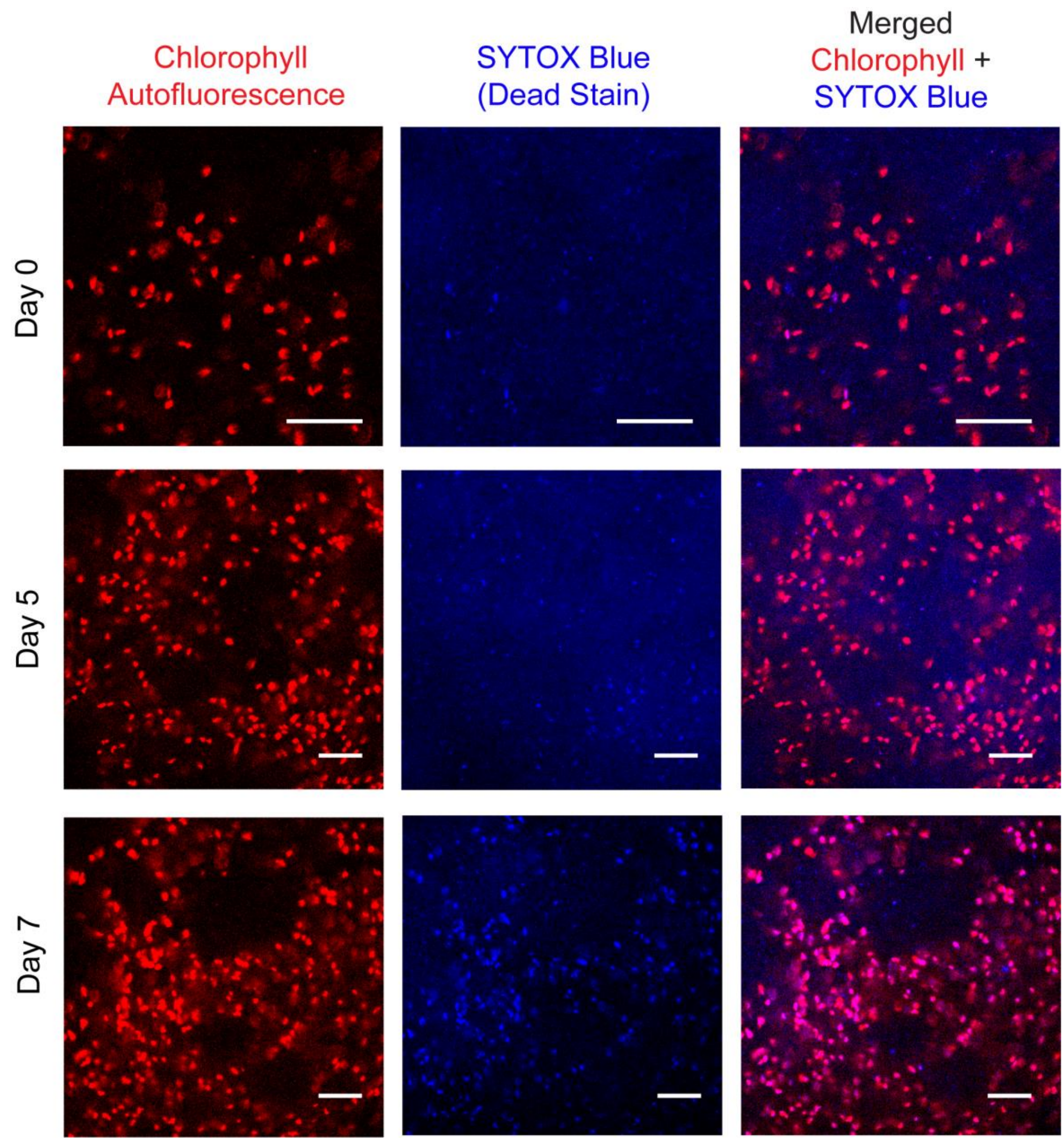

Supplementary Figure 3. Larger sized version of confocal images presented in Figure 2c (brightness enhanced uniformly across all panel, post processing) of hydrogel containing WT *S. elongatus* cells after printing and 0, 5, and 7 days of growth. Scale bar corresponds to 50 μm.

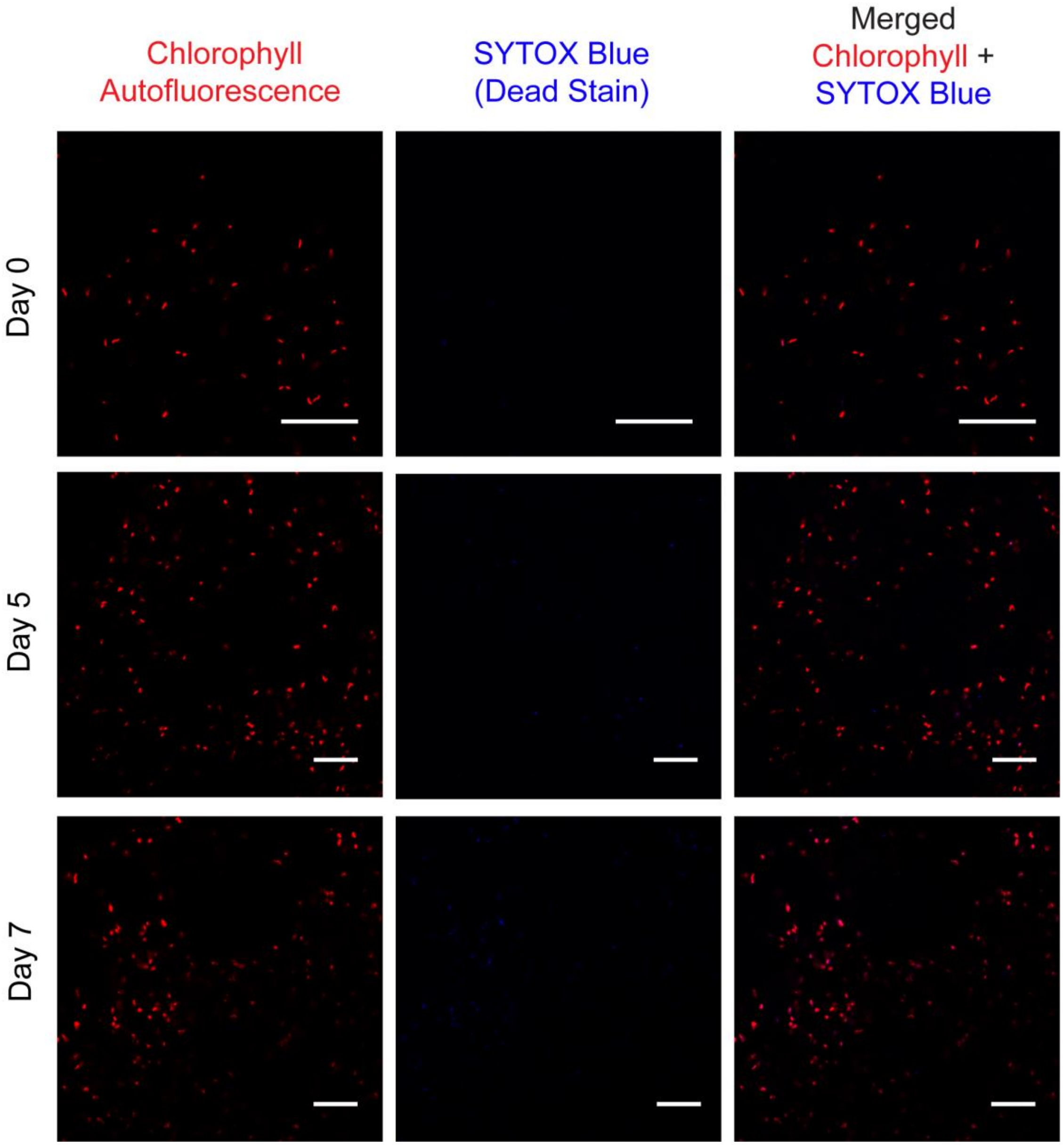

Supplementary Figure 4. Larger sized version of confocal images (raw data, without brightness enhancement) presented in Figure 2c of hydrogel containing WT *S. elongatus* cells after printing and 0, 5, and 7 days of growth. Scale bar corresponds to 50 µm.

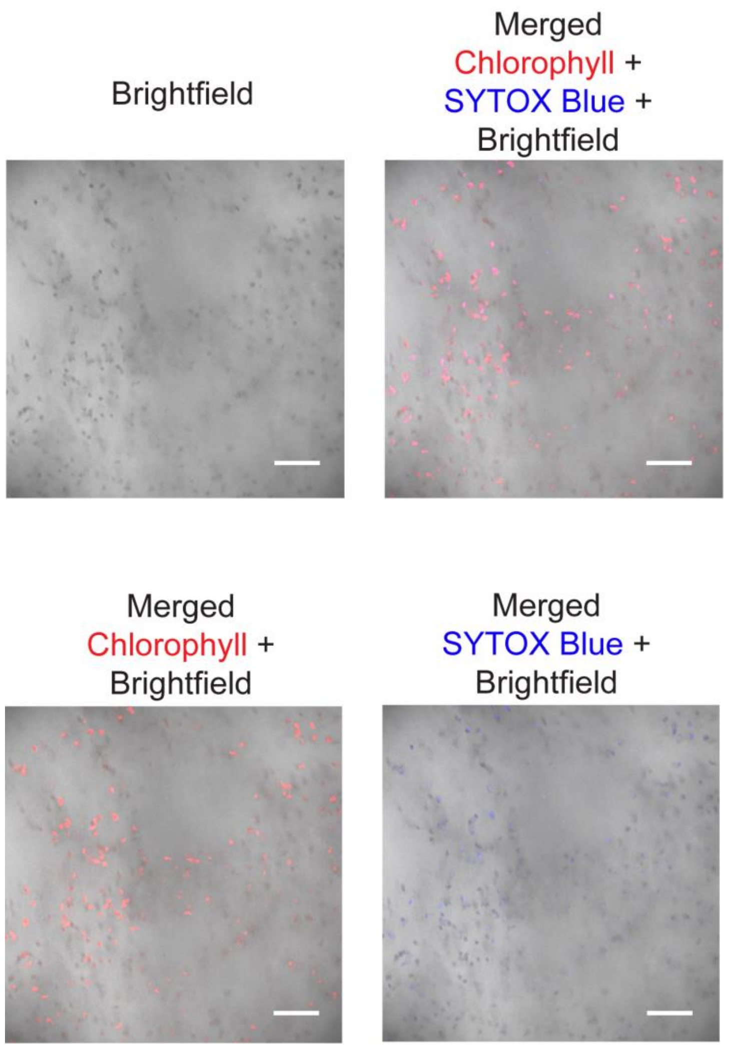

Supplementary Figure 5. Brightfield images from confocal data of hydrogel containing WT *S. elongatus* cells after printing and 7 days of growth, corresponding to Day 7 images of Supplementary Figure 4 above. Top left shows only brightfield image, top right shows superimposed chlorophyll + SYTOX blue + brightfield, bottom left shows superimposed chlorophyll + brightfield, and bottom right shows superimposed SYTOX blue + brightfield. Scale bar corresponds to 50 µm. Experiment was repeated thrice independently and gave similar results.

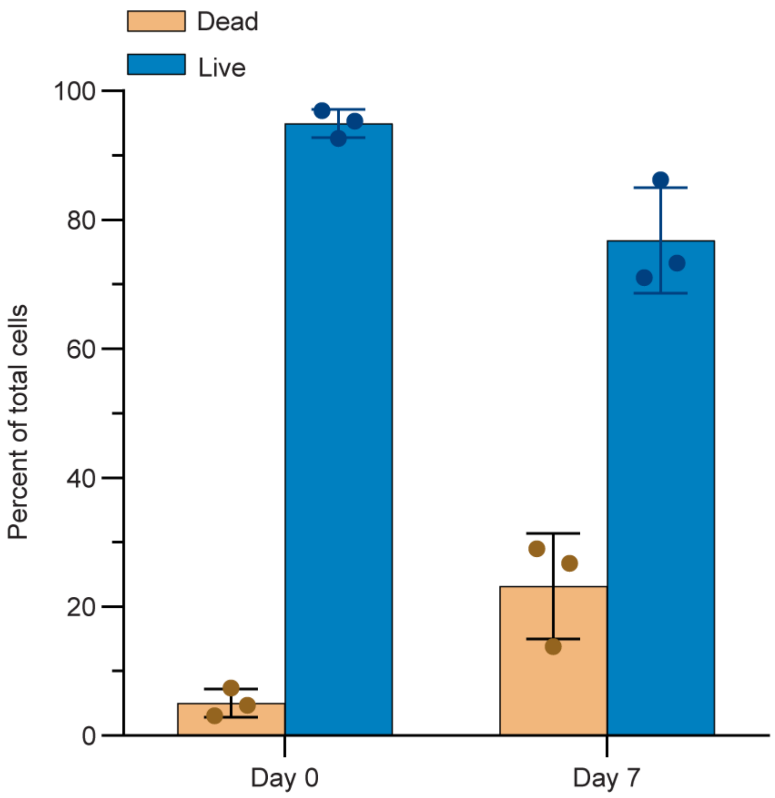

Supplementary Figure 6. Percentage cell viability as obtained from confocal image analysis from independent samples (n=3). Data are mean ± S.D.

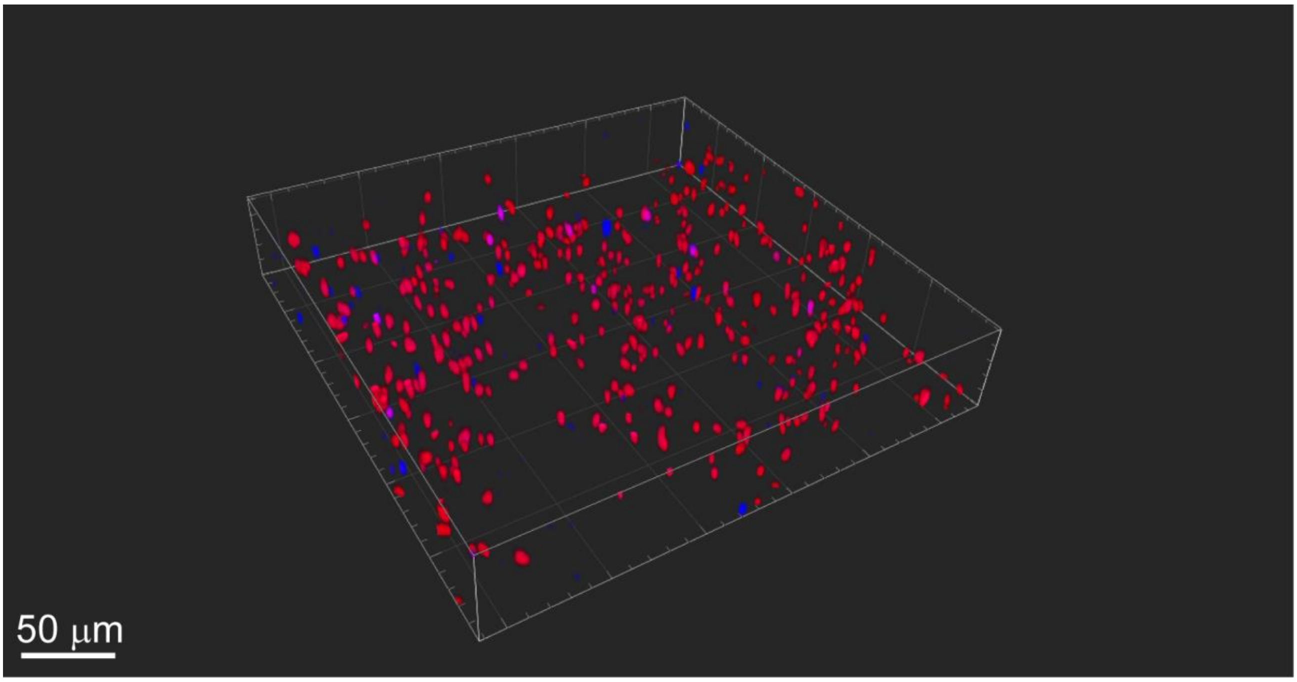

Supplementary Figure 7. Representative 3D reconstructed image from a confocal z stacked imaging data for the hydrogel containing WT *S. elongatus* cells. Axis X = Y = 289.21 µm and Z = 51.69 µm.

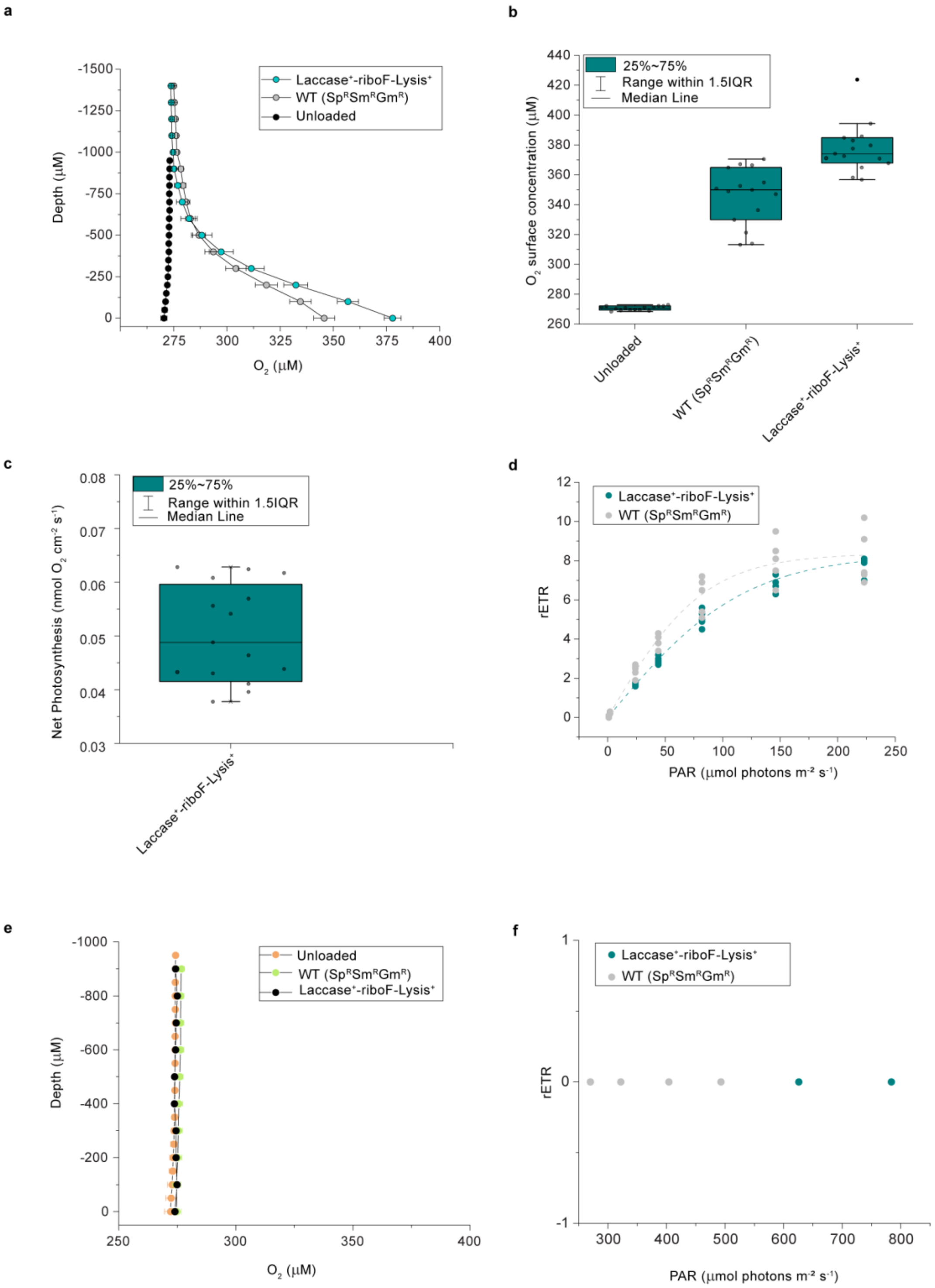

Supplementary Figure 8. O_2 microenvironment and photosynthetic activity of living hydrogels. a) O_2 microsensor profiles were performed from the hydrogel surface (depth = 0 µm) into the overlying water column for an unloaded hydrogel control, a hydrogel containing the WT($Sp^RSm^RGm^R$) (labeled Laccase$^-$-riboF-Lysis$^-$) strain and a hydrogel containing the Laccase$^+$-riboF-Lysis$^+$ strain. Data are means ± S.E.M. Measurements were taken from independent regions of each hydrogel (n=16 for Laccase$^+$-riboF-Lysis$^+$; n=15 for WT($Sp^RSm^RGm^R$) and Unloaded). b) Box plots with center lines showing the medians, boxes indicating the lower (25%) and upper (75%) quartiles, and whiskers indicating 1.5x interquartile range of O_2 concentrations measured at the surface of the hydrogel. Measurements were taken from independent regions of each hydrogel (n=15). c) Box plot with center lines showing the medians, boxes indicating the lower (25%) and upper (75%) quartiles, and whiskers indicating 1.5x interquartile range of net photosynthesis at an incident downwelling irradiance of 80 µmol photon m^{-2} s^{-1} for Laccase$^+$-riboF-Lysis$^+$ cells encapsulated in the hydrogel. Measurements were

taken from independent regions of a hydrogel (n=15). d) Relative electron transport (rETR) vs. photosynthetically active radiation (PAR; 400-700 nm) measured as rapid light curves for 5 replicate spots for each printed hydrogel of the mutant WT($Sp^R Sm^R Gm^R$) and Laccase$^+$-riboF-Lysis$^+$ strains. Dotted lines indicate non-linear curve fits according to the Platt photosynthesis model (see methods) ($r^2 > 0.95$). Measurements were taken from independent regions of each hydrogel (n=5). e) O_2 microsensor measurements during darkness. O_2 microsensor profiles were performed for an unloaded hydrogel control, a hydrogel containing the WT($Sp^R Sm^R Gm^R$) strain, and a hydrogel containing the Laccase$^+$-riboF-Lysis$^+$ strain. Measurements were taken from independent regions of each hydrogel (n=9 for Laccase$^+$-riboF-Lysis$^+$; n=15 for WT($Sp^R Sm^R Gm^R$) and n=14 for Unloaded). f) rETR vs. PAR data above 250 µmol photons m^{-2} s^{-1} showed full photoinhibition.

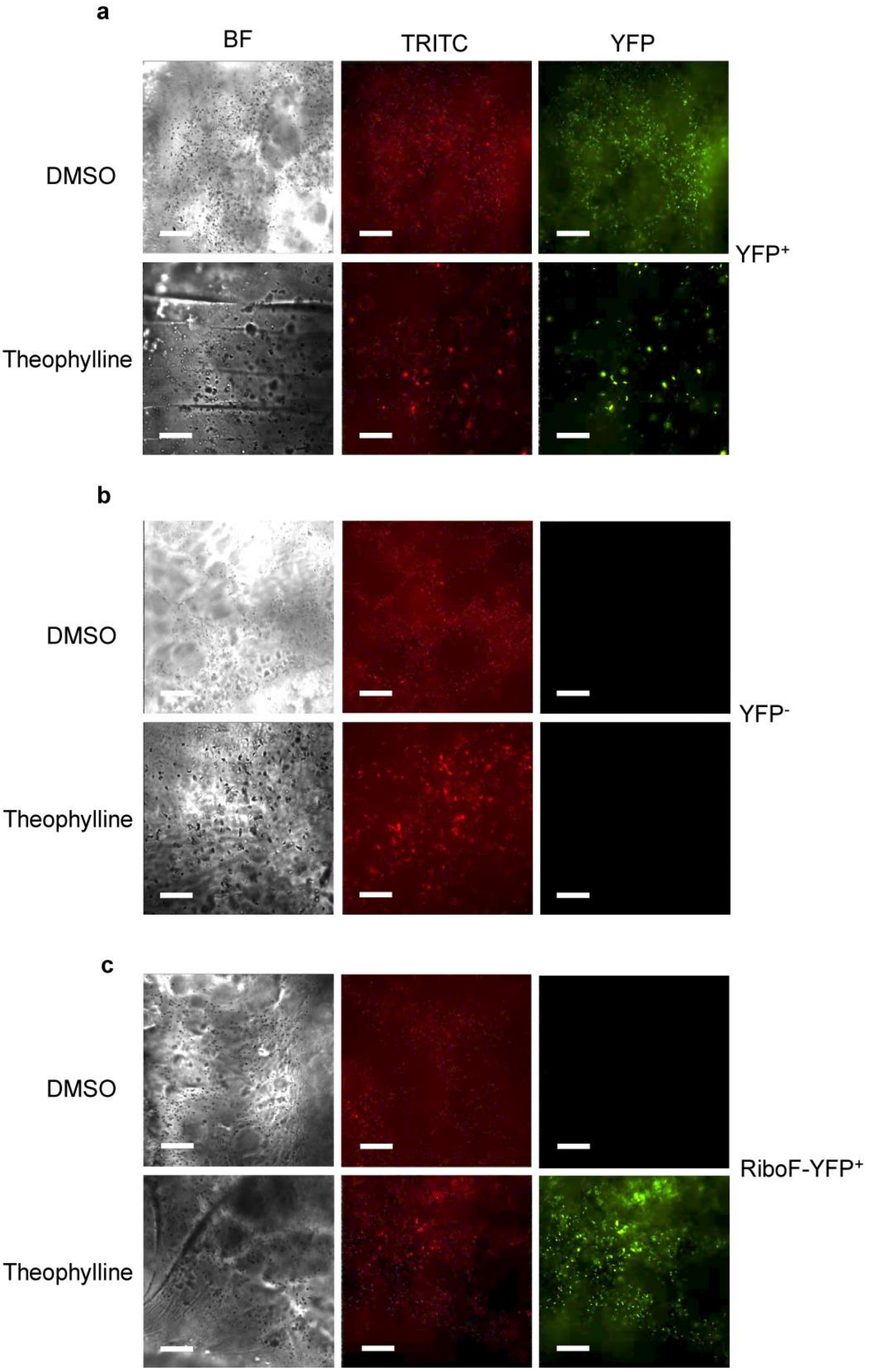

Supplementary Figure 9. Larger version of fluorescence microscopy images of hydrogels presented in Figure 3b containing a) YFP$^+$, b) YFP$^-$, and c) RiboF-YFP$^+$ strains. Images are shown for the hydrogels under brightfield (BF), TRITC, and YFP channels. Scale bar corresponds to 100 µm.

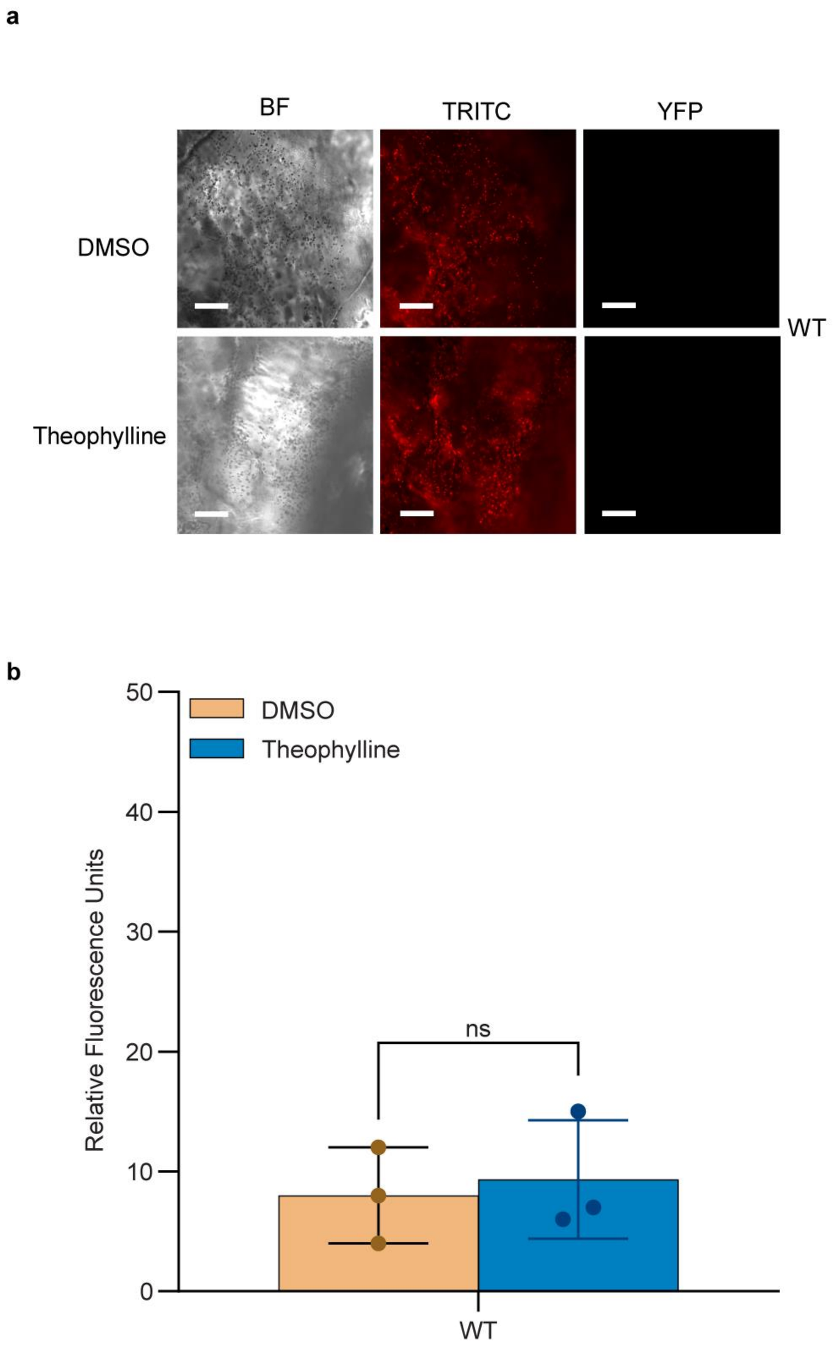

Supplementary Figure 10. YFP fluorescence signal from hydrogels and surrounding media containing WT strains. a) Representative fluorescence microscopy images of hydrogels containing WT *S. elongatus* cells. The hydrogels containing were supplemented with either 1% DMSO vehicle control or 1 mM theophylline. Images are shown for the hydrogels under brightfield (BF), TRITC, and YFP channels. Experiments were conducted with independent samples (n=3). Scale bar corresponds to 100 µm. b) YFP fluorescence measured from the BG-11 medium the ELM structure was incubated in. Experiments were conducted with independent samples (n=3). ns indicates not significant. P values were calculated using a two-tailed Student's t-test. Data are mean ± S.D.

a

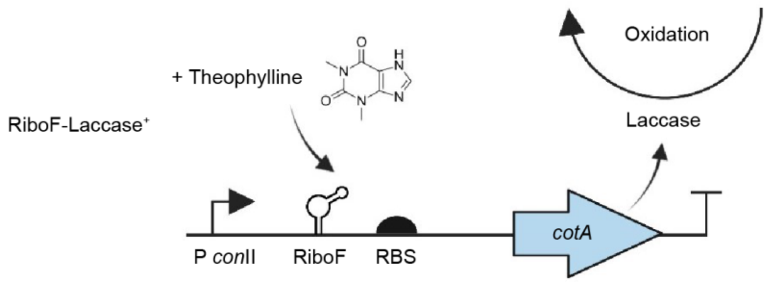

b

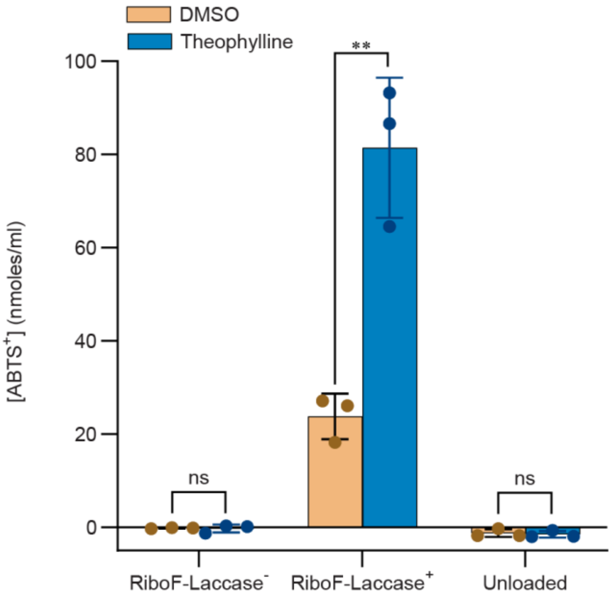

Supplementary Figure 11. ABTS activity from hydrogels containing RiboF-Laccase$^+$ strain. a) Schematic overview of the genetic circuit used in the construction of the RiboF-Laccase$^+$ strain. b) Oxidation of ABTS in reaction buffer by hydrogels containing strains RiboF-Laccase- control, RiboF-Laccase+, or unloaded hydrogel pre-incubated with either 1% DMSO or 1 mM theophylline. Experiments were conducted in triplicate from independent samples (n=3). ** and ns indicate P values ≤ 0.01, and not significant, respectively. P values were calculated using a two-tailed Student's t-test. The P values in **b** are (from left to right) 0.8464, 0.0032, and 0.7183. Data are mean ± S.D.

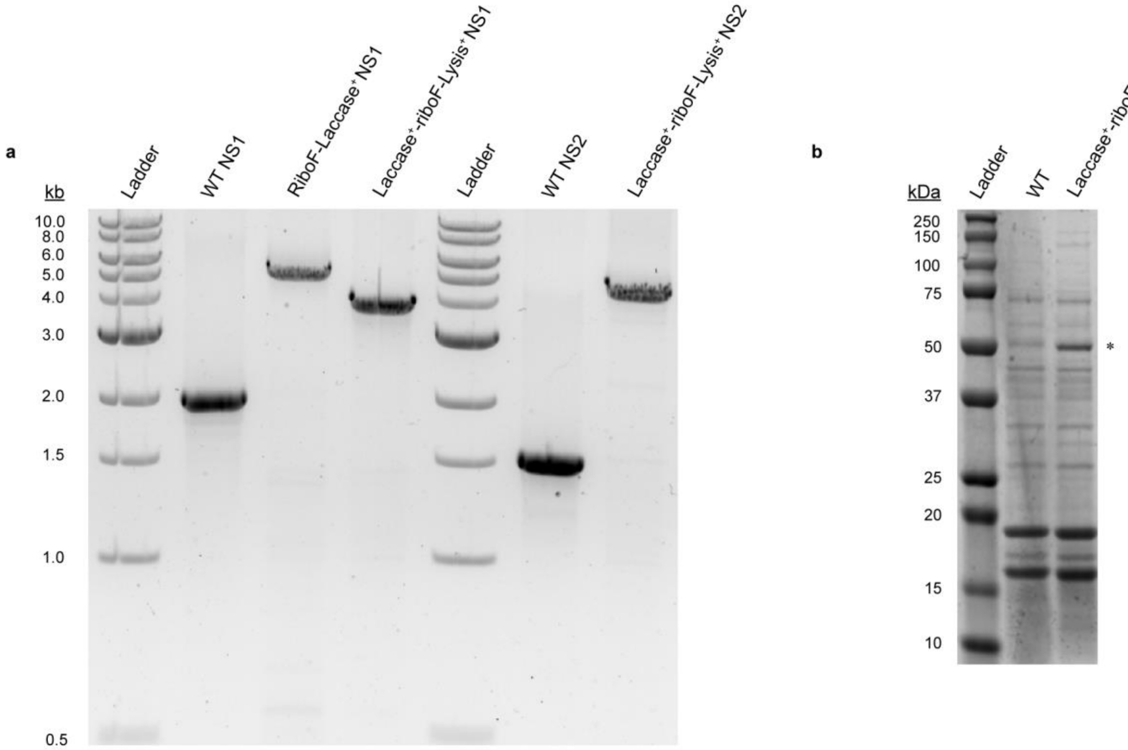

Supplementary Figure 12. Characterization of the strain Laccase^{+}-riboF-Lysis^{+}. a) Agarose gel showing the genotypic characterization of the Laccase^{+}-riboF-Lysis^{+} and RiboF-Laccase^{+} strains. Lane 1, standard 1-kb ladder (NEB); lane 2, PCR amplification of WT gDNA with primers surrounding neutral site 1; lane 3, PCR amplification of RiboF-Laccase^{+} gDNA with primers surrounding neutral site 1; lane 4, PCR amplification of Laccase^{+}-riboF-Lysis^{+} gDNA with primers surrounding neutral site 1; lane 5, standard 1-kb ladder (NEB); lane 6, PCR amplification of WT gDNA with primers surrounding neutral site 2; lane 7, PCR products of Laccase^{+}-riboF-Lysis^{+} gDNA with primers surrounding neutral site 2. b) SDS-PAGE of protein extracted from cultures of WT and Laccase^{+}-riboF-Lysis^{+} strains grown at 30 °C on an orbital shaker under 70 µmol photon m^{-2} s^{-1}, with the band corresponding to the predicted size of CotA indicated by an *. Each experiment was repeated twice independently and gave similar results.

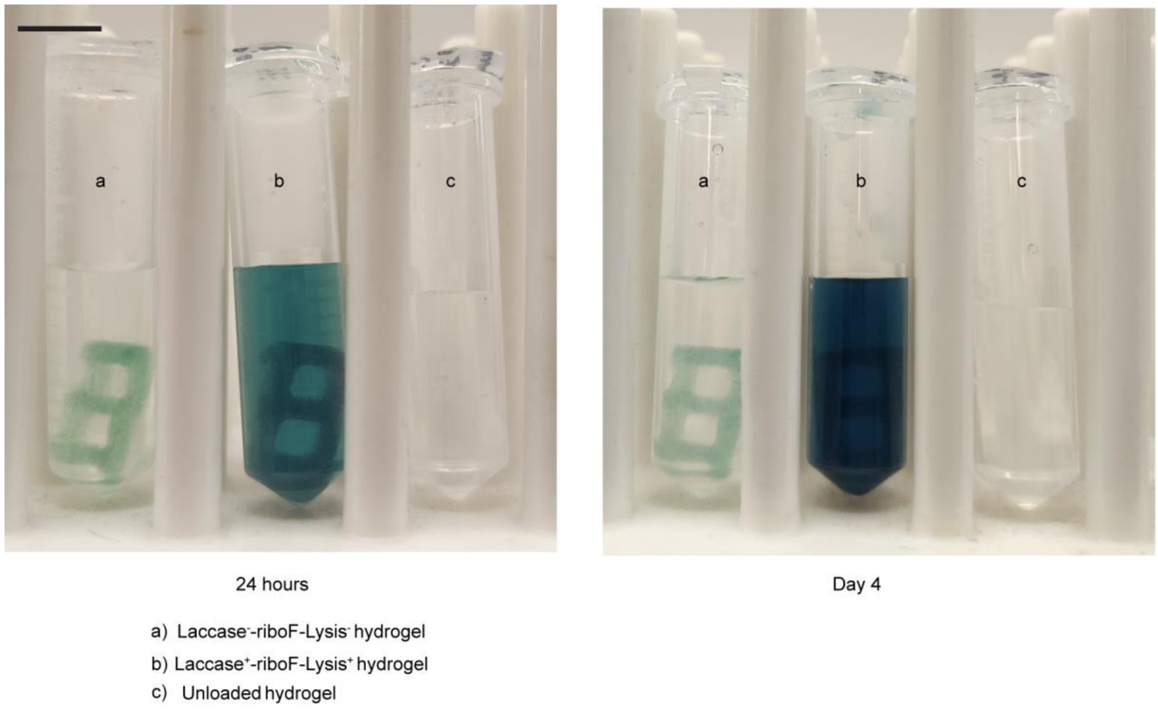

Supplementary Figure 13. Representative images of 3D printed patterns incubated in ABTS reaction buffer. The scale bar in upper left corresponds to 10 mm. Experiments were repeated thrice with independent samples (n=3).

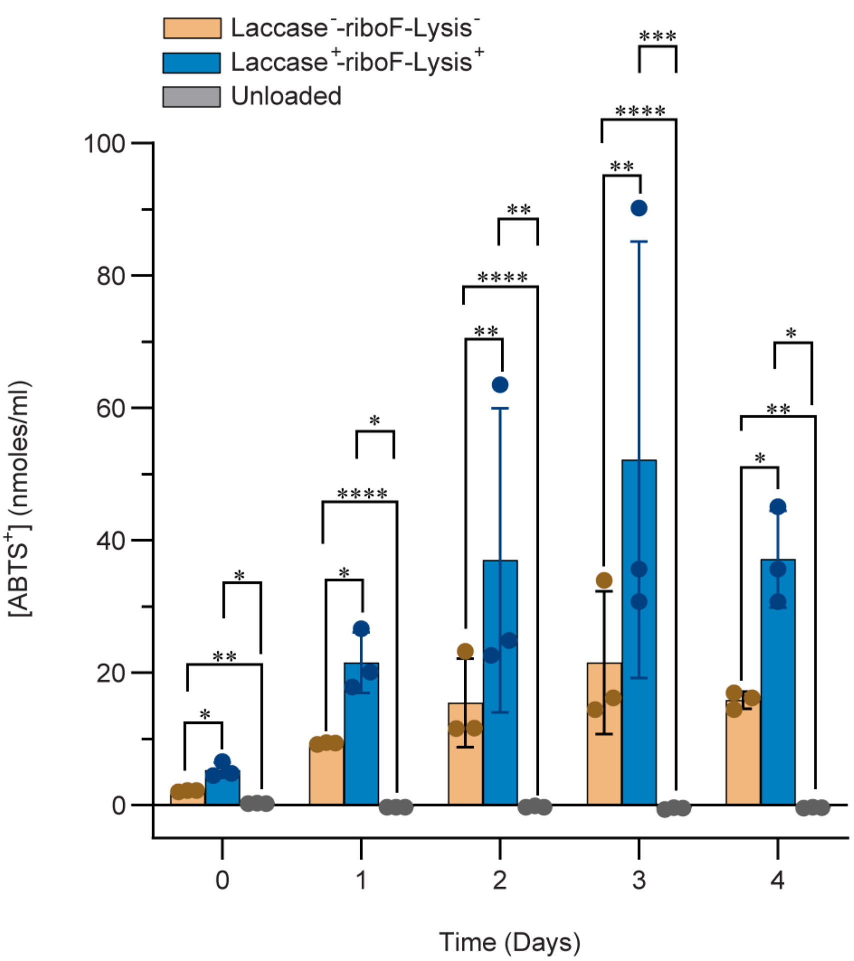

Supplementary Figure 14. Laccase activity of the supernatant of media surrounding hydrogels. Time-course of the oxidation of ABTS in reaction buffer with the addition of supernatant surrounding either the Laccase$^-$-riboF-Lysis$^-$, Laccase$^+$-riboF-Lysis$^+$ strain, or an unloaded hydrogel. Experiments were conducted in triplicate from independent samples (n=3). *, **, ***, and **** indicate P values ≤ 0.05, 0.01, 0.001 and 0.0001, respectively. P values were calculated using a two-tailed Student's t-test. The P values in **b** are (from left to right) 0.0408, 0.0011, 0.0174, 0.0436, 0.0001, 0.0143, 0.0057, 0.0000, 0.0105, 0.0076, 0.0000, 0.0009, 0.0333, 0.0022, and 0.0123. Data are mean ± S.D.

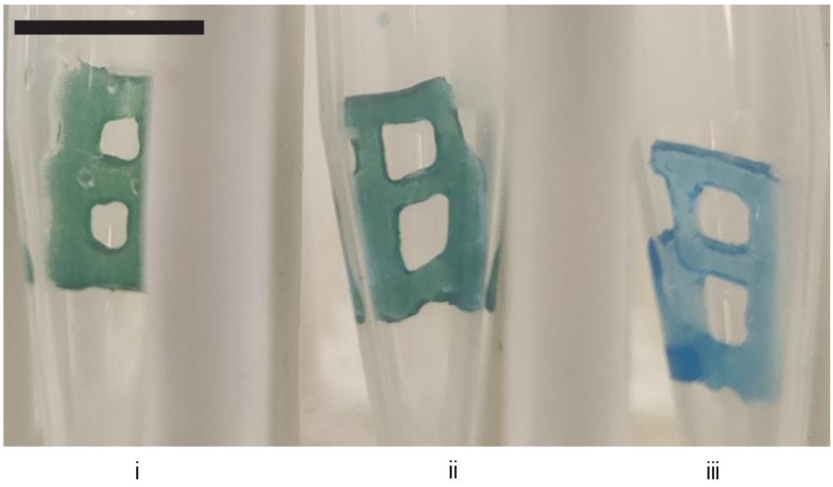

Supplementary Figure 15. Representative images of hydrogels separated from solutions after 10 days of incubation. Roman numerals indicate the addition of i) a hydrogel printed with Laccase^{+}-riboF-Lysis^{+}, ii) a hydrogel printed with Laccase^{-}-riboF-Lysis^{-}, iii) unloaded hydrogel. The scale bar in upper left corresponds to 10 mm. Experiments were repeated three times with independent samples (n=3).

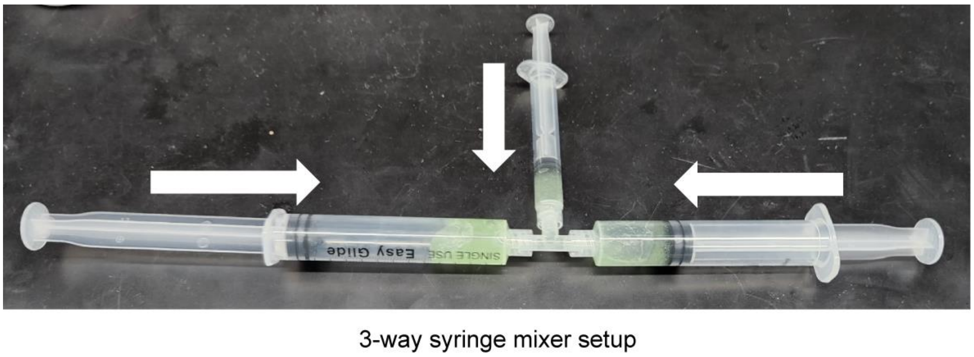

Supplementary Figure 16. In-house fabricated 3-way syringe mixer for bioink preparation.

Supplementary Note

1. Photosynthetic activity of strain Laccase$^+$-riboF-Lysis$^+$

Similar to the control strain, O_2 microsensors revealed a hyperoxic microenvironment on the surface of the living hydrogels that carried the embedded Laccase$^+$-riboF-Lysis$^+$ strain (**Supplementary Figure 8a & b**). O_2 concentrations were slightly higher for the Laccase$^+$-riboF-Lysis$^+$ hydrogel (mean=378 ± 4.2 µM SE); (Tukey post hoc p = <0.01) compared to the WT(Sp^{R}Sm^{R}Gm^R) hydrogel at an incident irradiance of 80 µmol photons m^{-2} s^{-1}. Net photosynthesis in the Laccase$^+$-riboF-Lysis$^+$ strain (**Supplementary Figure 8c**) was approximately 1.6-fold (0.05 nmol O_2 cm^{-2} s^{-1}) that of the WT(Sp^{R}Sm^{R}Gm^R) strain (0.031 nmol O_2 cm^{-2} s^{-1}, Tukey post hoc, $p < 0.01$). During darkness hydrogels that encapsulate the Laccase$^+$-riboF-Lysis$^+$ strain showed limited respiratory activity and O_2 concentrations were close to ambient seawater values as also observed for the control strain (**Supplementary Figure 8e**). Like the WT(Sp^{R}Sm^{R}Gm^R) *S. elongatus* strain, measurements of variable chlorophyll a fluorimetry for the Laccase$^+$-riboF-Lysis$^+$ strain was saturated between 200 – 250 µmol photons m^{-2} and indicative of low light adaptation with an irradiance at onset of saturation of about 120 µmol photons m^{-2} s^{-1} for the laccase mutant strain (**Supplementary Figure 8d**).

2. Construction of riboswitch-induced laccase strains and ABTS-Laccase activity testing for 3D printed constructs

In order to test the utility of the riboswitch regulatory mechanism for expression of a functional enzyme, we created a plasmid construct pAM5826 (**Supplementary Data 2, 4**) containing a theophylline-responsive riboswitch upstream of the *cotA* gene in pAM5825 and used to insert the cassette into the *S. elongatus* chromosome to produce strain RiboF-Laccase$^+$. **Supplementary Figure 11a** illustrates a schematic of the RiboF-Laccase$^+$ genetic circuit.

The surrounding solution of the hydrogel ELMs was tested for oxidative activity of the laccase enzyme against the substrate 2,2′-Azino-bis-(3-ethylbenzothiazoline-6-sulfonic acid) (ABTS). Briefly, after 5 days of growth, hydrogels containing strain RiboF-Laccase$^+$, RiboF-Laccase$^-$, and an unloaded-

control were transferred to fresh BG-11 medium supplemented with either 1 mM theophylline in DMSO or a 1% DMSO vehicle control and incubated for 72 hours. The hydrogels were then transferred to a buffer solution containing ABTS at a final concentration of 2 mM and incubated for 24 hours. The laccase activity was measured by quantifying concentrations of oxidized ABTS from the surrounding solution (**Supplementary Figure 11b)**. No oxidized ABTS was detected in either the RiboF-Laccase^{-}-containing or unloaded hydrogel samples. Levels of oxidized ABTS were 3.4-fold higher in RiboF-Laccase^{+} samples with theophylline induction relative to uninduced samples.

While YFP fluorescence was nearly undetectable in uninduced RiboF-YFP^{+} strains, moderate levels of laccase activity were detected in uninduced RiboF-Laccase^{+} samples. This difference is likely because of assay sensitivity due to the high activity of CotA and the low enzyme level required to oxidize ABTS. For future applications requiring tighter regulation of protein products, incorporating multiple riboswitches or pairing transcriptional regulatory systems with different riboswitches could be used to fine-tune expression.
