# Supplementary material for: Phenotypically complex living materials containing engineered cyanobacteria
Source: Nat Commun. 2023 Aug 7;14:4742. doi: 10.1038/s41467-023-40265-2 (PMC10406891; doi:10.1038/s41467-023-40265-2)
Supplement: Supplementary file 3 — Description of Additional Supplementary Files Document [file 41467_2023_40265_MOESM3_ESM.pdf]

Description of Additional Supplementary Files Document

Supplementary Movie

Supplementary Movie 1: 3D stacked images from confocal data showing cell viability with depth from sample hydrogel.

Supplementary Data

Supplementary Data 1: Different patterns of 3D printed alginate hydrogel along with their dimension and layer information of 3D printed gel patterns

Supplementary Data 2. Plasmids used in this study.

Supplementary Data 3. Primers used in this study.

Supplementary Data 4. Strains used in this study.
