# Supplementary material for: Phenotypically complex living materials containing engineered cyanobacteria
Source: Nat Commun. 2023 Aug 7;14:4742. doi: 10.1038/s41467-023-40265-2 (PMC10406891; doi:10.1038/s41467-023-40265-2)
Supplement: Supplementary file 6 — Reporting Summary [file 41467_2023_40265_MOESM6_ESM.pdf]

nature portfolio

Corresponding author(s): Susan S. Golden, Jonathan K. Pokorski

Last updated by author(s): Jul 19, 2023

Reporting Summary

Nature Portfolio wishes to improve the reproducibility of the work that we publish. This form provides structure for consistency and transparency in reporting. For further information on Nature Portfolio policies, see our Editorial Policies and the Editorial Policy Checklist.

Statistics

For all statistical analyses, confirm that the following items are present in the figure legend, table legend, main text, or Methods section.

n/a	Confirmed	
☐	☒	The exact sample size (*n*) for each experimental group/condition, given as a discrete number and unit of measurement
☐	☒	A statement on whether measurements were taken from distinct samples or whether the same sample was measured repeatedly
☐	☒	The statistical test(s) used AND whether they are one- or two-sided<br>*Only common tests should be described solely by name; describe more complex techniques in the Methods section.*
☒	☐	A description of all covariates tested
☒	☐	A description of any assumptions or corrections, such as tests of normality and adjustment for multiple comparisons
☐	☒	A full description of the statistical parameters including central tendency (e.g. means) or other basic estimates (e.g. regression coefficient) AND variation (e.g. standard deviation) or associated estimates of uncertainty (e.g. confidence intervals)
☐	☒	For null hypothesis testing, the test statistic (e.g. *F*, *t*, *r*) with confidence intervals, effect sizes, degrees of freedom and *P* value noted<br>*Give P values as exact values whenever suitable.*
☒	☐	For Bayesian analysis, information on the choice of priors and Markov chain Monte Carlo settings
☒	☐	For hierarchical and complex designs, identification of the appropriate level for tests and full reporting of outcomes
☐	☒	Estimates of effect sizes (e.g. Cohen's *d*, Pearson's *r*), indicating how they were calculated

Our web collection on statistics for biologists contains articles on many of the points above.

Software and code

Policy information about availability of computer code

Data collection	Rheological and viscosity measurements were collected with TRIOS software by TA Instruments. Confocal microscope images were recorded with LAS X software by Leica Microsystems. Scanning electron microscope images were recorded by the xT microscope control v13.9.1 in the FEI APREO FESEM, Thermoscientific instrument. O2 microsensor data was acquired with Pyroscience software (Profix v4.6) and Unisense software (Sensor Trace Suite (Logger v3.3)). Fluorescence microscopy images were recorded in NIS-Elements BR v5.41.01 software by Nikon. 3D printing Gcode was made using Cellink Heartware v2.4.1. All fluorescence and absorbance measurements were recorded by Tecan i-control v1.6.19.2 software by Tecan. Photographs were taken in phone camera.
Data analysis	Rheology and viscosity data was analyzed with TRIOS software. Fluorescence microscopy images were analyzed using NIS-Elements Viewer v5.21.00. Confocal data were analysed by LAS X, Fiji v2.9.0 and Imaris Viewer v9.9.0 software. Graphs were plotted using OriginPro v2021 and GraphPad Prism v9.0.

For manuscripts utilizing custom algorithms or software that are central to the research but not yet described in published literature, software must be made available to editors and reviewers. We strongly encourage code deposition in a community repository (e.g. GitHub). See the Nature Portfolio guidelines for submitting code & software for further information.

Data

Policy information about availability of data

All manuscripts must include a data availability statement. This statement should provide the following information, where applicable:

- Accession codes, unique identifiers, or web links for publicly available datasets
- A description of any restrictions on data availability
- For clinical datasets or third party data, please ensure that the statement adheres to our policy

All data supporting this study are included in this article, Supplementary Information, Supplementary Data and Source Data files. Source data are provided with this paper.

Research involving human participants, their data, or biological material

Policy information about studies with human participants or human data. See also policy information about sex, gender (identity/presentation), and sexual orientation and race, ethnicity and racism.

Reporting on sex and gender	N/A
Reporting on race, ethnicity, or other socially relevant groupings	N/A
Population characteristics	N/A
Recruitment	N/A
Ethics oversight	N/A

Note that full information on the approval of the study protocol must also be provided in the manuscript.

Field-specific reporting

Please select the one below that is the best fit for your research. If you are not sure, read the appropriate sections before making your selection.

☒ Life sciences ☐ Behavioural & social sciences ☐ Ecological, evolutionary & environmental sciences

For a reference copy of the document with all sections, see nature.com/documents/nr-reporting-summary-flat.pdf

Life sciences study design

All studies must disclose on these points even when the disclosure is negative.

Sample size	No statistical method was used to predetermine sample size. Sample size of n=3 independently transformed clones for all assays was chosen according to convention and due to high reproducibility of results.
Data exclusions	No data was excluded from this study.
Replication	Experiments were conducted in triplicate. Any experiments retested under identical conditions were successful.
Randomization	During experimentation, samples were organized in incubation chambers in random order.
Blinding	Randomly organized samples were measured discretely by either instrumentation of photograph, without space for operator influence.

Reporting for specific materials, systems and methods

We require information from authors about some types of materials, experimental systems and methods used in many studies. Here, indicate whether each material, system or method listed is relevant to your study. If you are not sure if a list item applies to your research, read the appropriate section before selecting a response.

Materials & experimental systems

n/a	Involved in the study
☒	☐ Antibodies
☒	☐ Eukaryotic cell lines
☒	☐ Palaeontology and archaeology
☒	☐ Animals and other organisms
☒	☐ Clinical data
☒	☐ Dual use research of concern
☒	☐ Plants

Methods

n/a	Involved in the study
☒	☐ ChIP-seq
☒	☐ Flow cytometry
☒	☐ MRI-based neuroimaging
